# Gender Differences in the Relationship between Type 2 Diabetes Mellitus and Employment: Evidence from the Korea Health Panel Study

**DOI:** 10.3390/ijerph17197040

**Published:** 2020-09-26

**Authors:** Jeung-Hee Kim, Weon-Young Lee, Song Soo Lim, Young Taek Kim, Yeon-Pyo Hong

**Affiliations:** 1 Division of Chronic Disease Control, Korea Disease Control and Prevention Agency, 187 Osongsaengmyeong 2-ro, Osong-eup, Heungdeok-gu, Cheongju-si, Chungcheongbuk-do 28159, Korea; lsgirl@hanmail.net; 2 Department of Food and Resource Economics, College of Life Science and Biotechnology, Korea University Graduate School, 145 Anamro, Seongbuk-gu, Seoul 02841, Korea; 3 Department of Preventive Medicine, College of Medicine, Chung-Ang University, 84 Heukseok-ro, Dongjak-gu, Seoul 06974, Korea; hyp026@cau.ac.kr; 4 Public Health Medical Service Office, Chungnam National University Hospital, 282 Munhwa-ro, Jung-gu, Daejeon 35015, Korea; ruyoung01@gmail.com

**Keywords:** gender difference, type 2 diabetes mellitus, employment, Korea

## Abstract

Previous studies have analyzed the impact of diabetes mellitus on labor market participation by men and women, but gender difference between type 2 diabetes mellitus (T2DM) and employment has not been the focus. This study aims to explore gender differences between T2DM and employment status. Data from the Korea Health Panel Study, 2013–2015 were analyzed by distinguishingT2DM and non-diabetes (*N* = 11,216). The empirical model was established and the generalized two-stage least squares (2SLS) was estimated, controlling for endogeneity. A family history of diabetes, as an instrumental variable, was related to an individual’s genetic predisposition to develop diabetes. The estimated results for the 2SLS showed the interaction effects between T2DM and employment. T2DM had a statistically significant and negative effect on employment for women only. The comparison with non-diabetes showed that women with T2DM had a lower probability of employment by 51.9% (*p* < 0.05). Exposing gender bias in employment suggests that healthcare policies and disease management programs for diabetic patients should adopt gender-specific remedies.

## 1. Introduction

Diabetes mellitus is a global health problem. The World Health Organization (WHO) estimated that 422 million adults were living with diabetes in 2014 as compared with 108 million in 1980. The global prevalence of diabetes in individuals aged >18 years has nearly doubled since 1980, increasing from 4.7% to 8.5%. Over the past decade, diabetes prevalence has more rapidly increased in low- and middle-income countries than in high-income countries [1]. The Centers for Disease Control and Prevention estimated that 23.1 million adults aged ≥18 years have diabetes, accounting for 7.2% of the U.S. population. The impact of the direct and indirect costs of diabetes on the national economy is shown by losses in gross domestic product(GDP) worldwide from 2011 to 2030, which amounts to USD 1.7 trillion [2]. The burden of diabetes in Korea is more problematic than in any other part of the world. Diabetes ranked fifth among the major causes of death in 2007 and sixth in 2017 [3]. According to the Korea National Health and Nutrition Examination Survey in 2017, the prevalence of diabetes in individuals aged ≥30 years was 10.4%. In terms of gender, the prevalence of diabetes in men (12.4%) was higher than that in women (8.4%) [4]. Furthermore, 90% to95% of all diabetes cases are likely to be type 2 diabetes mellitus (hereafter T2DM) [5]. The prevalence of T2DM has shown a rapid increase from <1% in 1960 to 6–9% by the end of the 1990s, and has been related to rapid urbanization, which introduced Western diet, lower levels of physical activity, and an increasingly obese population [6].

Relatively fewer studies have focused on the gender differences between T2DM and employment. Previous studies have analyzed the impact of diabetes mellitus on labor market participation by men and women [7]. Researches in Australia, Canada, and a number of European countries have examined the negative impact of diabetes on labor market participation outcomes [8,9,10]. In Australia, people with diabetes have a lower probability of being in the labor force for all age groups, and those out of the labor force have access to substantially lower incomes in retirement than non-diabetic people. In Canada, diabetic individuals with complications are twice as likely not to be in the labor force than their non-diabetics counterparts. In Europe, people diagnosed with diabetes have a 30% increase in the rate of labor force exit as compared with those without diabetes. These samples include women and men who are either randomly or consecutively selected as respondents.

Gendered inequalities vary with respect to employment and working conditions, with differences seen in occupations and economic activities, contract types, wages, access to labor rights and benefits, and working times [11,12] which can have beneficial or damaging effects on health. As indicated by the WHO, the unequal distribution of working conditions is a key contributor to social inequalities in health. Equal job opportunities for women and men and equal payment for the same job are still rarely seen around the world. In particular, women’s health inequalities have been related to an unequal distribution of social and family roles [13,14]. Gender roles play an important part in individuals’ roles, such that men rate their career role as more important than their family role as compared with women. Furthermore, sizeable gender gaps in employment and wages remain in virtually all countries, and women are underrepresented in high-paying occupations and leadership positions. In the USA, women earn, on average, 20% less than men on an hourly basis, and their employment rate is about 10 percentage points lower than that of men [15]. In the case of Korea, women continue to comprise a much greater proportion of the labor for rearing children, serving as the communication hub for family members, and managing family health. For this reason, women may be more vulnerable to stress both at home and in the workplace than men [16].

Therefore, this study examined the relationship between T2DM and employment status in terms of gender differences. To assess the causal relationships, we used the Korea Health Panel Study, an ongoing longitudinal study conducted in 2013–2015. We estimated the generalized two-stage least squares (2SLS), controlling for endogeneity. Family history of diabetes, as an instrumental variable (IV), was related to an individual’s genetic predisposition to develop diabetes [17,18,19].

## 2. Materials and Methods

### 2.1. Data Source

The Korea Health Panel Study (KHPS Version 1.4) is an official statistical investigation that has been conducted annually since 2008 by the Korea Institute for Health and Social Affairs and National Health Insurance Service. In selecting respondents nationwide, the KHPS uses stratified multistage probability sampling according to region and residence from the 2005 Korea Census. The objectives of the investigation were to identify the factors influencing the use of health care services, health expenditures, and financial resources, and to provide basic information on policy implementation by continuing to observe health care trends. The data collected were on demographic socioeconomic characteristics (e.g., age, sex, marital status, education level, health insurance type, employment status, household generation, and household income), chronic illness, and medical use, including medication, emergency service, and admission, of 7000–8000 household members who agreed to complete in-home face-to-face interviews in 16 cities/provinces [20].

To examine the association between T2DM and employment decisions, we used data from 5521 households and 16,247 individuals in 2013, 5284 households and 15,263 individuals in 2014, and 5098 households and 14,344 individuals in 2015.

### 2.2. Study Design

**Figure 1:**
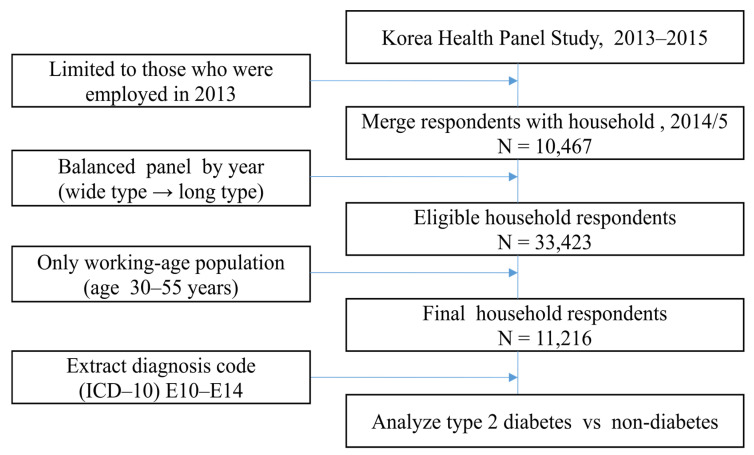
Study design.

The key dependent variable of the KHPS was the change in employment status of an individual who was employed in 2013, as indicated in Figure 1. In two consecutive years, respondents participated in a household survey, which was classified by household code. We identified 10,467 consecutive households with the parameters assessed, including presence of chronic illness, employment, and household income. In econometrics analysis, the structure of panel data should be changed from wide to long type [21]. Thus, our dataset reshaped the structure of the data and contained a balanced panel without annual time gaps. Among 33,423 household respondents, the sample was restricted to those of working age at 30–55 years, with the average age being 46 years. The final sample size was determined as 11,216 household respondents.

### 2.3. Variable Selection

Longitudinal studies considered the International Classification of Diseases10 (ICD-10) of survey participants when indicating the type of chronic disease. We extracted respondents who met the ICD–10 code for diabetes diagnosis, E10–E14, from the KHPS data. Specifically, to avoid obtaining biased estimates of the effects of diabetes on labor market outcomes, we distinguished between T2DM and non-diabetes cases. Hence, respondents with missing values of the chronic disease code and those who were diagnosed without consideration for the combination of co-morbidities (e.g., hypertension, hyperlipidaemia, arthropathy, and ischemic heart disease), which could lead to bias in estimates attributable to diabetes, were excluded.

The employment variable was obtained from a question that asked if the individuals were working for 12 months before the interview. Sociodemographic variables included age, marital status, education, school graduation, and national health insurance. Education variables were characterized as no formal education, elementary school, middle-high school, 2–4 years of college, or graduate school. Because Korea has adopted universal insurance coverage since 1989, national health insurance variables have been classified into two types, which are consistent with the characteristics of employment status and medical aids [22]. The household region variable included respondents who lived in 16 cities/provinces that were categorized as urban or rural regions. Household generation was defined as the number of generations in a household, consisting of the first generation, second generation, and so on. Household head indicated whether the individual was the householder or the head of a family. The owner of the house variable indicated whether or not the individual owned the house. Yearly household income (KRW10,000) was the total income of all household individuals who lived in a house. Subjective health satisfaction variables were categorized as normal-good or bad [23,24]. The variable definitions are given in Table 1.

We used family history of diabetes as an IV, which would explain the association between T2DM and employment outcomes as a potentially endogenous variable in the analyses. Employment participation could influence unmeasured personal characteristics, such as age at diabetes onset, Westernized diet, and exercise. Personal traits could influence employment; motivation could influence lifestyle choice; and healthy lifestyles could decrease the risk of developing diabetes [25,26]. Thus, unobserved factors need to be controlled for because T2DM could lead to endogeneity bias with respect to employment status. Our study used the sibling with diabetes variable as an IV extracted from the KHPS data (e.g., diabetic grandparent, diabetic parents, and diabetic sibling), to control for unobserved factors of T2DM.

### 2.4. Empirical Model

To analyze the characteristics of the panel data, we tested the study hypotheses using the Hausman test for fixed effects (correlated errors) vs. random effects. The null hypothesis was not rejected because the *p*-value was 1.000 in men but 0.392 in women. Therefore, the preferable model was the random effects model at the 5% level of confidence [21]. Two models were subsequently estimated. Model I assumed that estimates of T2DM were exogenous and indicated the average effect of T2DM relative to those without diabetes. Model II was estimated with the generalized two-stage least squares (2SLS) using a diabetic sibling as an IV [27]. Thus, we used Model I for probit regression and Model II for2SLS regression.

Model I:

Y_it_* = *a* + *β*_1_X_*it*_ + *β*_2_Type2_*it*_ + *u*_*i*_ + *e*_*it*_

$$\left\{ \begin{array}{c} Y_{it}=1\;\mathrm{If}\;Y_{it}{}^{*}>0\\ Y_{it}=0\mathrm{Otherwise}\mathrm{if}Y_{it}{}^{*}\leq 0,u_{i}\sim N(0,\sigma_{u^{2}}) \end{array}\right.$$

where Y_it_* > 0 and Y_it_* ≤ 0 represent that individual *i* is employed or otherwise, respectively. The variable Y*_it_* is dichotomous, which is equal to 1 if individual *I* employed during the survey period. X*_it_* is a vector of sociodemographic (age, marital status, education, school graduation, and national health insurance), household variables (household region, household generation, household head, owner of the house, and yearly household income) and subjective health satisfaction variables. Type 2*_it_* is an indicator variable, which is equal to 1 if individual *i* has reported a diagnosis of T2DM and 0 if otherwise. Moreover, u*_i_* is a stochastic error term.

Model II:

$$\mathrm{Type}2_{it}=\delta_{1}+\delta_{2}X_{it}+\delta_{3}Z_{it}+\nu_{i}+\varepsilon_{it}\;Y_{it}=\;a\;+\;\beta_{1}X_{it}\;+\;\beta_{2}\hat{T}\mathrm{ype}2_{it}+u_{i}+e_{it}$$

Type 2*_it_* is dichotomous, which is equal to 1 if individual *i* has reported a diagnosis of T2DM and 0 if otherwise during the survey period. X*_it_* is a vector of sociodemographic (age, marital status, education, school graduation, and national health insurance), household variables (household region, household generation, household head, owner of the house, and yearly household income), and subjective health satisfaction variables. Z*_it_* is a vector of family history of diabetes as an IV. The model assumes that *u_i_* is normally distributed. Particularly, the assumption of *Cov*(X*_it_*, *u_i_*) = 0 is needed for a 2SLS estimator to satisfy a consistent estimator. For ameliorating the endogeneity bias, the second equations were calculated estimates of T2DM ($\hat{T}$ype2*_it_*) using the IV, such as sibling with diabetes.

## 3. Results

Our final sample size was 11,216, comprising 5466 men and 5750 women. Table 2 shows the descriptive statistics by gender with and without T2DM. The sociodemographic characteristics of household respondents who were employed in 2013 included the changes in employment status and working conditions in 2014–2015. Considering the population with T2DM in the two consecutive years, we found that 94.2% of men were employed as compared with 77.9% of women.

In men, the average age of individuals with T2DM was 46.6 years, and that of those without diabetes was 45.6 years. The T2DM group marked a relatively higher proportion of older respondents (*p* < 0.05) and those living in multigenerational households (*p* < 0.01) as compared with non-diabetes groups. Ages between 40 and 55 years comprised 86.5% of T2DM patients and 81.2% of individuals with non-diabetes. For household generation, T2DM had a means of 2.12 (SD = 0.48) and non-diabetic, 2.04 (SD = 0.4). However, we found no statistical difference between individuals with T2DM and those without diabetes with respect to the following variables: employment status, marital status, education, school graduation, national health insurance, household region, household head, owner of the house, yearly household income, and subjective health satisfaction.

For women, the average age of individuals with T2DM was 46.1 years, and that of those without diabetes was 45.6 years. The comparison with non-diabetes groups showed that the T2DM group participants were more likely to live in urban areas and to be in multigenerational households (*p* < 0.01) but were less likely to be household heads and recorded a lower subjective health satisfaction (*p* < 0.05). Living in urban areas comprised 57.7% of the T2DM patients and 41.2% of the individuals with non-diabetes. For household generation, T2DM had a means of 2.13 (SD = 0.51) and non-diabetes, 2.0 (SD = 0.4). Household head represented 3.7% of T2DM patients and 8.7% of non-diabetes individuals. Respondents between normal and good in subjective health satisfaction comprised 80.9% of T2DM patients and 87.1% of non-diabetes individuals. However, no statistical difference was found between the T2DM group and non-diabetes groups with respect to the following: employment status, age, marital status, education, school graduation, national health insurance, owner of the house, and yearly household income.

Table 3 presents the effects of T2DM on employment status by gender using probit regression and 2SLS regression. Model I did not reveal significant impacts of T2DM on employment status among men, whereas according to Model II there was a significant positive impact of T2DM on employment decisions (*p* < 0.05). Model II also showed that school graduation, national health insurance, household generation, household head, owner of the house, yearly household income, and subjective health satisfaction had significant positive effects, whereas age had significant negative effects on the developing T2DM and employment (*p* < 0.01). Thus, men with T2DM were significantly more likely to be employed than men without diabetes, with an estimated increment in employment probability of 25.3%.

For women, Model I showed that T2DM had no significant impact on employment decisions, whilst Model II showed a significant negative impact of T2DM on employment status (*p* < 0.05). Model II also identified marital status and school graduation as negative factors, whereas age and national health insurance were identified as positive drivers in the T2DM and employment relationship (*p* < 0.01). Therefore, women with T2DM were significantly less likely to be employed than women without diabetes, with an estimated reduction in employment probability of 51.9%.

## 4. Discussion

Previous studies have analyzed the impact of diabetes mellitus on labor market participation by men and women, but gender difference between T2DM and employment has not been the focus. Thus, we examined gender differences between T2DM and employment status using the Korea Health Panel Study. We estimated the generalized two-stage least squares (2SLS), controlling for unobserved factors of T2DM and used an instrumental variable (IV), such as a sibling with diabetes. Our finding demonstrated the interaction effects between T2DM and employment. T2DM had a statistically significant and negative effect on employment in women only.

This finding is consistent with previous studies. Gender gaps in employment have been attributed to women having to care for the socially weak population, including childbearing and childrearing. Meanwhile, the employment status of men is less affected, owing to the influence of labor market productivity on the probability of men’s labor force [12,26]. Indeed, studies have consistently indicated that women were more involved in family roles and had higher expectations of those roles than men [13,14]. Similar to the IV methodology, our analyses presented the impact of diabetes on employment status, estimated after controlling for the existence of endogeneity using a diabetic sibling as an IV. Latif [18] used family history of diabetes as an IV and found that diabetes had a significant negative impact on employment in women. Minor [19] analyzed the effects of T2DM separately and used individuals’ biological mother as an IV; the results showed that women with T2DM were significantly less likely to be employed at almost 50% as compared with healthy individuals.

These findings have significant implications for policy and practice. Public health policies to prevent chronic diseases must do more to protect the employment of patients with T2DM. Particularly, women seem to be vulnerable to leaving employment owing to their larger day-to-day household responsibilities, including childcare and housework. Moreover, understanding gender differences in T2DM can be useful for policymakers who are tasked with reducing the gender gap in employment and researchers who want to analyze deeply these decision factors. Korea should consider developing tailored and evidence-based prevention and disease management programs for diabetic patients using gender-specific remedies [28,29,30].

Meanwhile, our study has several limitations. First, insufficient IVs could lead to inconsistent results on diabetes. The number of IVs used in the estimation must be equal to or greater than the number of endogenous explanatory variables [21]. Further research using more IVs could be driven by the accurate identification of samples. Second, our data did not include potential discrimination for T2DM in the workplace. Owing to the nature of our data, we could not identify employment patterns. Indeed, previous studies have shown that diabetes per se is not a source of stigma [9,31]. Third, the nature of employment was not considered in our study. In short, having a blue-or white-collar job and being fully or partially employed could be dependent on gender. These factors need to be considered in further studies.

Nevertheless, our study has the following main strengths. We used the KHPS and analyzed nationally representative dataset. Our method provided the opportunity to account for time-invariant unobserved characteristics. We also distinguished T2DM using theICD-10 diagnosis code and excluded respondents with missing values or a combination of co-morbidities (e.g., hypertension, hyperlipidaemia, arthropathy, and ischemic heart disease). Thus, our study could analyze purely the impact of T2DM using family history of diabetes as an IV, by controlling for unobserved factors, such as personal traits, motivation, and healthy lifestyle, with respect to employment status.

## 5. Conclusions

This study explored the relationship between T2DM and employment status in terms of gender differences by using a nationally representative dataset (KHPS Version 1.4). Our results showed that the interaction effects between T2DM and employment. T2DM had a statistically significant and negative effect on employment of women only. Women, who are in an inferior position in the labor market, are the main provider of childcare and domestic work. The negative influence on T2DM is more marked for women as compared with men. To our knowledge, this study is the first to report the impact of employment decision on T2DM by gender in Korea. Exposing gender bias in employment suggests that healthcare policies and disease management programs for diabetic patients should adopt gender-specific remedies. Further research needs to investigate the factors underlying the persistent gender differences in performing family responsibilities.

## Figures and Tables

**Table 1:** Definition of variables.

Variable	Variable Name	Definition
Employment status	Employment	1 if employed in each year from2013to2015; 0 otherwise
Diabetes mellitus	T2DM	1 if diagnosed with diabetes code E10–E14; 0 if non-diabetic
Family history of diabetes	Sibling diabetes	1 if sibling with diabetes; 0 otherwise
Sociodemographic	Age	1 if 30–39 years; 2 if 40–49 years; 3 if 50–55 years
	Marital status	1 if married; 0 if divorced/widowed/separated
	Education	1 if no formal education; 2 if elementary school; 3 if middle–high school; 4 if 2–4 years of college; 5 if graduate school
	School graduation	1 if yes; 0 otherwise
	National health insurance	1 if health insurance; 0 if medical aids
Households	Household region	1 if area of residence is urban; 0 if area of residence is rural
	Household generation	number of generations: first generation, secondgeneration, etc.
	Household head	1 if yes; 0 otherwise
	Owner of the house	1 if house ownership; 0 otherwise
	yearly household income	the total income of all household ones (in KRW10,000)
Subjective health satisfaction		1 if normal-good; 0 if bad

**Table 2:** Descriptive statistics by gender with and without type 2 diabetes mellitus (T2DM) in the Korea Health Panel Study, 2013–2015.

Variables	Men (*N* = 5466)					Women (*N* = 5750)				
	T2DM (*N* = 207)		Non-Diabetic (*N* = 5259)			T2DM (*N* = 163)		Non-Diabetic (*N* = 5587)		
	*n* %		*n* %		*p*	*n*%		*n* %		*p*
Employment status										
Employed	195	94.2	4952	94.2		127	77.9	4492	80.4	
Unemployed	12	5.8	307	5.8		36	22.1	1095	19.6	
Mean ± (SD)	0.942 ± (0.234)		0.941 ± (0.234)		0.981	0.779 ± (0.416)		0.804 ± (0.396)		0.431
Age										
30–39 years	28	13.5	993	18.9		25	15.3	1129	20.2	
40–49 years	97	46.9	2538	48.3		84	51.5	2549	45.6	
50–55 years	82	39.6	1728	32.9		54	33.1	1909	34.2	
Mean ± (SD)	46.570 ± (6.260)		45.551± (6.733)		<0.05	46.055 ± (6.495)		45.635 ± (6.782)		0.436
Marital status										
Married	157	75.9	4036	76.7		138	84.7	4617	82.6	
Divorced/widowed/separated	50	24.2	1223	23.3		25	15.3	970	17.4	
Mean ± (SD)	0.758 ± (0.429)		0.767 ± (0.422)		0.764	0.846 ± (0.361)		0.826 ± (0.378)		0.501
Education										
No formal education	2	0.9	6	0.1		3	1.8	22	0.4	
Elementary school	4	1.9	83	1.6		9	5.5	255	4.6	
Middle-high school	93	44.9	2366	44.9		92	56.4	3260	58.4	
2–4 years of college	104	50.2	2501	47.6		54	33.1	1891	33.9	
Graduate school	4	1.9	303	5.8		5	3.1	159	2.9	
Mean ± (SD)	3.502 ± (0.622)		3.572 ± (0.631)		0.116	3.300 ± (0.703)		3.341 ± (0.629)		0.412
School graduation										
Yes	198	96.6	5021	95.6		153	95.6	5390	96.9	
No	7	3.4	232	4.4		7	4.4	175	3.1	
Mean ± (SD)	0.965 ± (0.182)		0.955 ± (0.205)		0.492	0.956 ± (0.205)		0.968 ± (0.174)		0.382
National health insurance										
Health insurance	202	97.6	5127	97.5		158	96.9	5354	95.8	
Medical aids	5	2.4	132	2.5		5	3.1	233	4.2	
Mean ± (SD)	0.975 ± (0.153)		0.974 ± (0.156)		0.932	0.969 ± (0.172)		0.958 ± (0.199)		0.486
Household region										
Urban	97	46.9	2230	42.4		94	57.7	2302	41.2	
Rural	110	53.1	3029	57.6		69	42.3	3285	58.8	
Mean ± (SD)	0.468 ± (0.500)		0.424 ± (0.494)		0.204	0.576 ± (0.495)		0.412 ± (0.492)		<0.01
Household generation										
Mean± (SD)	2.120 ± (0.482)		2.038 ± (0.399)		<0.01	2.128 ± (0.510)		1.995 ± (0.444)		<0.01
Household head										
Yes	162	78.3	4285	81.5		6	3.7	484	8.7	
No	45	21.7	974	18.5		157	96.3	5103	91.3	
Mean ± (SD)	0.782 ± (0.413)		0.814 ± (0.388)		0.244	0.036 ± (0.188)		0.086 ± (0.281)		<0.05
Owner of the house										
Yes	134	64.7	3607	68.6		105	64.4	3833	68.6	
No	73	35.3	1652	31.4		58	35.6	1754	31.4	
Mean ± (SD)	0.647 ± (0.478)		0.685 ± (0.464)		0.242	0.644 ± (0.480)		0.686 ± (0.464)		0.257
Yearly household income										
Mean ± (SD)	5650.908 ± (2785.4)		5542.178 ± (4238.2)		0.714	5860.578 ± (2998.6)		5629.309 ± (3513.1)		0.406
Subjective health satisfaction										
Normal-good	169	89.4	4380	90.8		127	80.9	4765	87.1	
Bad	20	10.6	446	9.2		30	19.1	703	12.9	
Mean ± (SD)	0.894 ± (0.308)		0.907 ± (0.289)		0.534	0.808 ± (0.394)		0.871± (0.334)		<0.05
Sibling diabetes			-					-		
Yes	10	4.8				4	6.2			
No	197	95.2				61	93.9			
Mean ± (SD)	0.048 ± (0.214)					0.061 ± (0.242)				

Note: Standard deviation in parentheses.

**Table 3:** Effect of T2DM on employment status by gender using probit regression and two-stage least squares(2SLS) regression.

Variables	Men				Women			
	Model I		Model II		Model I		Model II	
	Coef. (*z*)	*p*	Coef. (*z*)	*p*	Coef. (*z*)	*p*	Coef. (*z*)	*p*
T2DM	0.164 (0.61)	0.544	0.253 (2.04)	<0.05	−0.086 (−0.38)	0.705	−0.519 (−2.22)	<0.05
Age	−0.022 (−2.46)	<0.05	−0.003 (−3.92)	<0.01	0.049 (6.73)	<0.01	0.009 (3.59)	<0.01
Marital status	0.109 (0.62)	0.535	0.014 (1.04)	0.298	−1.160 (−7.45)	<0.01	−0.141 (−2.62)	<0.01
Education	0.002 (0.03)	0.979	0.005 (0.74)	0.460	−0.067 (−0.93)	0.353	−0.002 (−0.07)	0.944
School graduation	0.305 (1.65)	<0.1	0.066 (3.73)	<0.01	−0.385 (−1.78)	<0.1	−0.206 (−2.56)	<0.01
National health insurance	0.749 (3.23)	<0.01	0.099 (4.28)	<0.01	1.251 (6.41)	<0.01	0.212 (2.96)	<0.01
Household region	−0.079 (−0.78)	0.435	−0.005 (−0.70)	0.481	−0.463 (−5.73)	<0.01	0.005 (0.16)	0.869
Household generation	0.356 (2.66)	<0.01	0.035 (3.73)	<0.01	0.115 (1.29)	0.197	0.006 (0.21)	0.836
Household head	2.017 (8.23)	<0.01	0.230 (14.62)	<0.01	−0.134 (−0.66)	0.508	0.015 (0.21)	0.832
Owner of the house	0.245 (2.11)	<0.05	0.023 (2.94)	<0.01	−0.267 (−3.15)	<0.01	−0.028 (−0.95)	0.340
Yearly household income	0.00009 (4.96)	<0.01	2.44× 10^−6^(3.37)	<0.01	0.00002 (2.13)	<0.05	1.89 × 10^−6^ (0.50)	0.616
Subjective health satisfaction	0.628 (4.90)	<0.01	0.056 (5.59)	<0.01	0.129 (1.34)	0.181	0.048 (1.45)	0.148

Note: Model I uses probit regression and Model II uses 2SLS regression.
